# TROP2 Is Uniformly Expressed in Primary Prostate Cancer but Frequently Reduced in Recurrent Disease

**DOI:** 10.3390/cancers18142359

**Published:** 2026-07-22

**Authors:** Jonathan Jeutner, Ronald Simon, Maximilian Lennartz, Sarah Minner, Eike Burandt, Fiete Gehrisch, Nina Schraps, Martina Kluth, Guido Sauter, Henning Plage, Jacob Schmidt, Natalia Gorbokon, Florian Viehweger, Hans Heinzer, Alexander Haese, Thorsten Schlomm, Markus Graefen, Stefan Steurer, Ria Schlichter, Christian Bernreuther, David Dum, Andreas Luebke, Bernhard Ralla, Neele Heckmann

**Affiliations:** 1 Department of Urology, Charité—University Medical Center Berlin, 10117 Berlin, Germany; jonathan.jeutner@charite.de (J.J.); thorsten.schlomm@charite.de (T.S.); 2 Institute of Pathology, University Medical Center Hamburg-Eppendorf, 20246 Hamburg, Germany; r.simon@uke.de (R.S.); m.kluth@uke.de (M.K.); g.sauter@uke.de (G.S.); cbernreuther@uke.de (C.B.); luebke@uke.de (A.L.); neele.heckmann@uksh.de (N.H.); 3 Department of General, Visceral and Thoracic Surgery, University Medical Center Hamburg-Eppendorf, 20246 Hamburg, Germany; 4 Martini-Clinic, Prostate Cancer Center, University Medical Center Hamburg-Eppendorf, 20246 Hamburg, Germany; heinzer@uke.de (H.H.);

**Keywords:** TROP2, TACSTD2, prostate cancer, tissue microarray, immunohistochemistry, recurrence, ERG, biochemical recurrence

## Abstract

**Background**: TROP2 (TACSTD2) is a therapeutic target of antibody–drug conjugates and is broadly expressed in epithelial malignancies. Data on TROP2 protein expression in large, clinically annotated prostate cancer cohorts, particularly in recurrent disease, remain limited. **Methods**: TROP2 immunohistochemistry was performed on a prostate cancer tissue microarray comprising 17,747 primary radical prostatectomy specimens and 258 recurrent prostate cancer samples. TROP2 staining data were compared with clinicopathological parameters, biochemical recurrence and ERG status. **Results**: Among 12,807 interpretable primary prostate cancers, TROP2 staining was detectable in all cases and considered strong in 94.5%, moderate in 3.5%, and weak in 2.0%. Reduced TROP2 was statistically associated with high pT stage (*p* = 0.0011) and high Gleason grade (*p* < 0.0001), but absolute differences were small. Reduced TROP2 expression levels were unrelated to PSA recurrence in the entire cohort (*p* = 0.0787) but statistically linked to early PSA recurrence in ERG positive cancers (*p* = 0.0165). In four different scenarios of multivariate analyses, reduced TROP2 expression did not show an unequivocal independent prognostic role. As compared to primary tumors, TROP2 was markedly reduced among 250 evaluable recurrent cancers (strong 57.6%, moderate 31.6%, weak 8.4%, negative 2.4%; *p* < 0.0001 vs. primary cancers). Reduced TROP2 was also significantly more frequent in recurrent cancers than in the subgroup of high-grade primary tumors (Gleason ≥ 4+4; strong 57.6% vs. 90.2%; *p* < 0.0001). **Conclusions**: TROP2 is consistently expressed at high levels in primary prostate cancer but often reduced in recurrent disease. While uniform expression in primary tumors supports TROP2 as a therapeutic target in prostate cancer, reduced expression in many recurrent cancers highlights the potential need for TROP2 expression measurement as a predictive test in this subgroup.

## Simple Summary

Prostate cancer remains one of the most common cancers in men, and new treatment strategies are urgently needed, particularly for patients whose disease has returned after initial therapy. One promising approach involves drugs that selectively deliver toxic compounds to cancer cells by targeting a protein called TROP2, which is found on the surface of many tumor cells. To evaluate whether this approach is suitable for prostate cancer, we examined TROP2 protein levels in tissue samples from more than 17,000 prostate cancer patients. We found that TROP2 is present at high levels in virtually all primary prostate cancers, making it an attractive therapeutic target. However, in patients with recurrent disease—where new treatment options are most urgently needed—TROP2 levels were substantially lower in many cases. These findings suggest that measuring TROP2 in tumor tissue may help identify which patients with recurrent prostate cancer are most likely to benefit from TROP2-targeting therapies.

## 1. Introduction

The Trophoblast cell surface antigen 2 (TROP2), also known as tumor-associated calcium signal transducer 2 (TACSTD2) is a transmembrane glycoprotein encoded by the TACSTD2 gene on chromosome 1p32 [1,2]. TROP2 has been implicated in epithelial cell biology and cancer-related processes including cell self-renewal, proliferation, and malignant transformation [3,4,5]. In normal tissues, TROP2 expression is largely restricted to epithelial cells, whereas overexpression has been described across a wide range of carcinomas, supporting its broad potential as a therapeutic target candidate. The clinical translation of this concept has progressed furthest through the development of antibody–drug conjugates (ADCs) directed against TROP2, most notably sacituzumab govitecan (SG; Trodelvy™, Gilead Sciences), an ADC consisting of a humanized anti-TROP2 antibody linked to SN-38, the active metabolite of irinotecan [6].

SG has shown clinically meaningful activity in several cancer types and has received regulatory approval for defined subsets of metastatic triple-negative breast cancer and metastatic urothelial carcinoma [7,8,9,10,11,12,13,14]. For prostate cancer, preclinical experiments have demonstrated efficacy of SG in TROP2 expressing prostate cancer cell lines [4] but so far few prostate cancer patients have been treated with SG in published datasets. In the phase I/II IMMU-132-01 basket trial, one of 11 patients with castration-resistant prostate cancer (CRPC) had a complete response [8]. In a separate open-label phase II trial in patients with metastatic CRPC (mCRPC), SG resulted in a 6-month radiographic progression-free survival rate of 52% (14/27) overall [15]. In an individual-use series of nine heavily pretreated patients with TROP2-positive aggressive variant/neuroendocrine prostate cancer, SG achieved partial remission in 44% and stable disease in 33%, with disease control rates of 78% and 44% at 3 and 6 months, respectively [16].

The clinical benefit of SG therapy has been independent of TROP2 expression levels in several studies, and data on the potential predictive value of TROP2 expression measurement for response prediction remain inconsistent across tumor entities [7,11,17]. For prostate cancer, prior immunohistochemical studies have reported heterogeneous positivity rates in primary, hormone-naïve prostate cancer ranging from 30 to 66% and variable associations with tumor aggressiveness and outcome in studies analyzing cohorts of 74–1153 cancers [18,19,20]. Moreover, data on TROP2 expression in recurrent prostate cancer are scarce, although this setting is of utmost clinical relevance for treatment escalation and for the development of targeted strategies.

To further assess the role of TROP2 in prostate cancer and its potential as a drug target, we investigated TROP2 protein expression by immunohistochemistry (IHC) on a large prostate cancer tissue microarray (TMA) comprising samples from more than 17,000 primary radical prostatectomy specimens and >250 recurrent prostate cancers by using a previously validated TROP2 IHC assay.

## 2. Materials and Methods

**Patients:** Radical prostatectomy (RP) specimens from 17,747 patients treated between 1992 and 2015 at the Department of Urology and the Martini Clinic, University Medical Center Hamburg-Eppendorf, were included in this study. In addition, 258 recurrent prostate cancer samples were represented in the same tissue microarray (TMA) cohort, resulting in a total of 18,005 tissue spots. Clinical follow-up information was available for 14,464 patients with a median follow-up of 48 months (range 1–275 months; Table 1). The recurrent prostate cancer cohort consisted of archived tumor samples collected during clinical routine. Detailed clinical annotation of recurrent cases (e.g., prior therapies) was not consistently available for all samples.

Postoperative prostate-specific antigen (PSA) was measured at regular intervals. Biochemical recurrence was defined as a postoperative PSA ≥ 0.2 ng/mL followed by a subsequent increase. Histopathological parameters (pT stage, Gleason grade, lymph node status, and surgical margin status) were retrieved from clinical and pathology records. In addition to conventional Gleason grading, quantitative Gleason grading (percentage of Gleason pattern 4) was recorded as described previously [21]. TMA construction has been described in detail [22]. In brief, one 0.6 mm tissue core was taken from a cancer-containing paraffin block from each RP specimen and arrayed into 40 TMA blocks (144–522 tissue spots per block). The molecular database attached to this TMA contained results on ERG protein expression in 12,789 and ERG break-apart fluorescence in situ hybridization (FISH) analysis in 7036 (expanded from [23,24]). Archived diagnostic left-over tissues were used in accordance with local regulations (HmbKHG, §12(1)) and approved by the local ethics committee (Ethics Commission Hamburg, WF-049/09). All work was conducted in accordance with the Declaration of Helsinki.

**Immunohistochemistry (IHC):** Freshly cut TMA sections were immunostained on one day and in one experiment. Slides were deparaffinized with xylene, rehydrated through a graded alcohol series and exposed to heat-induced antigen retrieval for 5 min in an autoclave at 121 °C in pH 7.8 Tris-EDTA-Citrate (TEC) buffer. Endogenous peroxidase activity was blocked with Dako REAL Peroxidase-Blocking Solution (Agilent Technologies, Santa Clara, CA, USA; #S2023) for 10 min. The primary antibody specific for TROP2 (recombinant rabbit monoclonal, clone MSVA-733R, MS Validated Antibodies, Hamburg, Germany) was applied at 37 °C for 60 min at a dilution of 1:150. The bound antibody was then visualized using the Dako REAL EnVision Detection System Peroxidase/DAB+, Rabbit/Mouse kit (Agilent Technologies, Santa Clara, CA, USA; #K5007) according to the manufacturer’s directions. The sections were counterstained with hemalaun. For tumor tissues, the percentage of positive neoplastic cells was estimated, and the staining intensity was semi-quantitatively recorded (0, 1+, 2+, 3+). For statistical analyses, the staining results were categorized into four groups. Tumors without any staining were considered negative. Tumors with 1+ staining intensity in ≤70% of tumor cells or 2+ intensity in ≤30% of tumor cells were considered weakly positive. Tumors with 1+ staining intensity in >70% of tumor cells, 2+ intensity in 31–70%, or 3+ intensity in ≤30% of tumor cells were considered moderately positive. Tumors with 2+ intensity in >70% or 3+ intensity in >30% of tumor cells were considered strongly positive.

**Statistics:** Statistical calculations were performed with JMP 16 software (SAS Institute Inc., Cary, NC, USA). Contingency tables and the χ^2^-test were performed to search for associations between TROP2 immunostaining and tumor phenotype. Time to biochemical recurrence was analyzed by Kaplan–Meier estimates and compared by the log-rank test. Cox regression models were used to test for independent prognostic role of parameters.

## 3. Results

**Technical issues:** A total of 18,005 tissue spots were present on the TMA, including 17,747 primary radical prostatectomy specimens and 258 samples from recurrent prostate cancer. Of the primary tumor spots, 12,807 (72.2%) were interpretable. Non-informative spots lacked unequivocal tumor cells or contained no tissue. Among recurrent prostate cancer samples, 250 (96.9%) were evaluable.

**Figure 1:**
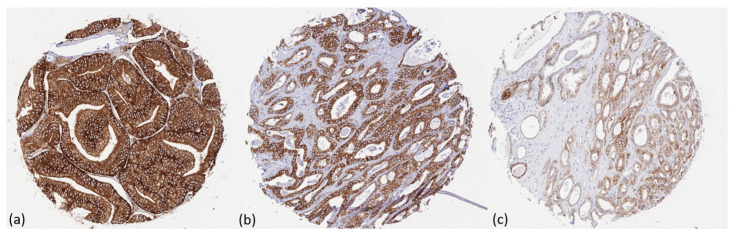
Representative examples of TROP2 immunostaining in prostate cancer. (**a**) Strong, (**b**) moderate and (**c**) weak staining.

**Figure 2:**
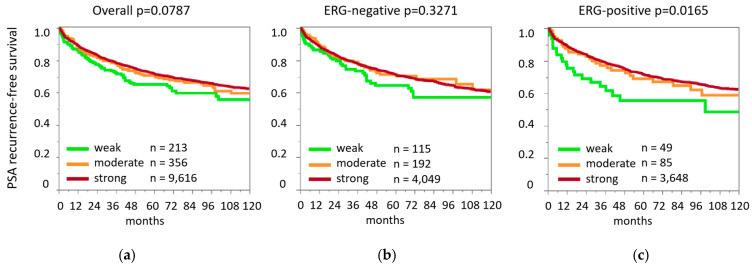
Association of TROP2 expression with biochemical (PSA) recurrence in prostate cancer. Kaplan–Meier analysis of time to PSA recurrence stratified by TROP2 immunostaining categories (weak, moderate, strong) in (**a**) all cancers (*p* = 0.0787), (**b**) ERG-negative cancers (*p* = 0.3271), and (**c**) ERG-positive cancers (*p* = 0.0165).

**TROP2 expression in primary prostate cancer**: TROP2 staining was detectable in all 12,807 interpretable primary prostate cancers. Staining was categorized as strong in 94.5%, moderate in 3.5%, and weak in 2.0% of tumors (Figure 1; representative examples of weak, moderate, and strong staining across multiple tissue microarray spots are provided in Figures S1–S3). Reduced TROP2 expression was significantly associated with advanced pT stage (*p* = 0.0011) and high traditional and quantitative Gleason grade (*p* < 0.0001), although absolute differences were small (Table 2). For example, strong TROP2 expression was observed in 95.3% of pT2 tumors and in 93.2% of pT3b–pT4 tumors. Across Gleason categories, strong expression decreased from 95.2% in Gleason 3+3 to 90.2% in Gleason ≥ 4+4 cancers. No significant associations were found with lymph node status (*p* = 0.24), preoperative PSA (*p* = 0.10), or surgical margin status (*p* = 0.16). Reduced TROP2 expression was not significantly related to early PSA recurrence (*p* = 0.0787; Figure 2).

**TROP2 expression and ERG status:** Because the TMPRSS2:ERG fusion is the most common molecular alteration in prostate cancer and defines a major molecular subclass, we examined whether TROP2 expression and its prognostic associations differ by ERG status. Among tumors with interpretable TROP2 staining, ERG FISH data were available for 5307 and ERG IHC data for 9925 cases. Although the absolute differences were only minimal—TROP2 staining was present in 98.8% of TMPRSS2:ERG fusion-positive and in 97.5% of fusion-negative cancers—this difference reached statistical significance (*p* < 0.0001). Reduced TROP2 expression was significantly linked to early PSA recurrence in ERG-positive cancers (*p* = 0.0165) but not in ERG-negative cancers (*p* = 0.3271).

**Figure 3:**
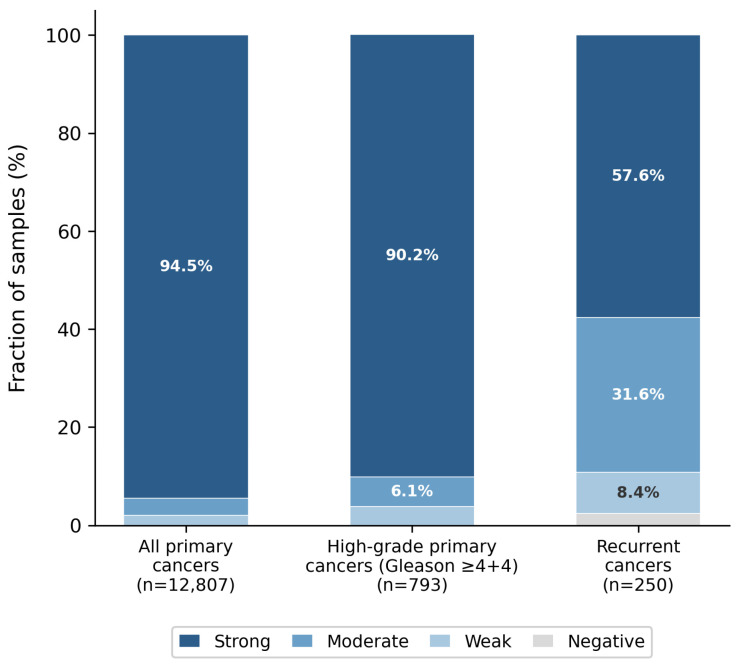
TROP2 expression in primary and recurrent prostate cancer. Stacked bar chart showing the proportional distribution of TROP2 immunostaining categories (strong, moderate, weak, negative), drawn to scale, in all primary cancers (*n* = 12,807), high-grade primary cancers (Gleason ≥ 4+4; *n* = 793), and recurrent cancers (*n* = 250).

**TROP2 expression in recurrent prostate cancer:** TROP2 expression was markedly lower in recurrent than in primary prostate cancers (*p* < 0.0001). Among 250 recurrent prostate cancers, TROP2 staining was strong in 57.6%, moderate in 31.6%, weak in 8.4%, and negative in 2.4% of cases (Figure 3). Because tumor grade and recurrence represent distinct dimensions—a primary tumor may be of high grade and stage without being recurrent—we performed a grade-matched comparison to isolate the effect of recurrence from that of grade. To determine whether reduced expression in recurrent disease merely reflects the higher grade typically seen in advanced tumors, TROP2 expression in recurrent cancers was compared to high-grade primary tumors (Gleason ≥ 4+4). Strong TROP2 staining remained significantly less frequent in recurrent cancers (57.6%) than in high-grade primary tumors (90.2% strong, *p* < 0.0001; Figure 3).

**Multivariate Cox regression analyses:** To evaluate the independent prognostic impact of TROP2 expression, four predefined multivariable Cox regression models were calculated for the overall cohort as well as for ERG-negative and ERG-positive cancers (Table 3). Scenario 1 included all available postoperative parameters (pT stage, pN status, surgical margin status, preoperative PSA, and prostatectomy Gleason grade). Scenario 2 used the same parameters but excluded pN due to non-standardized indication and the extent of lymph node dissection. Two additional models were designed to approximate the preoperative setting: Scenario 3 included preoperative PSA, clinical tumor stage (cT), and prostatectomy Gleason grade, whereas Scenario 4 used biopsy Gleason grade instead of prostatectomy Gleason grade. In 12 analyses involving the entire cohort and the subgroups of ERG positive and ERG negative cancers, there were three scenarios for which borderline independent associations (*p* < 0.05/>0.01) were seen (Table 3). Considering also the large number of tumors involved in these multivariate analyses, our data indicate that TROP2 expression does not provide clinically important independent prognostic information beyond established clinicopathological parameters.

## 4. Discussion

The successful analysis of 12,807 primary prostate cancers revealed TROP2 positivity in all cases, with the vast majority (94.5%) showing strong expression. This observation is in line with the 98.4% positivity found in 247 prostate cancer cases in the only previous study which also aimed to distinguish a subgroup of completely negative cancers. In this study by Dum et al., TROP2 expression had been evaluated in 18,563 samples from 150 different tumor types by using the same assay as in our study [25]. Our TROP2 IHC assay had been extensively validated by the authors according to the recommendations of the international working group for antibody validation (IWGAV) by comparison of IHC findings obtained on 76 different normal tissue types with RNA expression data and with staining results by an independent second antibody and RNA expression data [26].

Independent transcriptomic data support these findings. In a large analysis of metastatic castration-resistant prostate cancer (n = 634), TACSTD2 mRNA was broadly expressed across luminal and basal adenocarcinoma subtypes but was expressed at lower levels in neuroendocrine prostate cancer, the most dedifferentiated phenotype [27]. Consistent with the near-uniform TROP2 protein expression observed in our primary tumors, publicly available transcriptomic data from The Cancer Genome Atlas likewise indicate substantial TACSTD2 expression in primary prostate adenocarcinoma [28]. These orthogonal, RNA-based observations, generated on a different platform and in independent cohorts, are in line with both principal findings of our study: high TROP2 expression in primary disease and its reduction with progression toward dedifferentiated, treatment-resistant states.

While TROP2 is extensively expressed in both normal prostate epithelium and in most primary prostate cancers, our data demonstrated a tendency towards a reduced expression going along with tumor progression in pT stage and Gleason grade. These observations are in contrast to data from several earlier studies which have not distinguished TROP2 negative cases but described various proportions of tumors with low and high TROP2 positivity in considerably smaller cohorts (148–1153 cases), so that their dichotomized “high-versus-low” comparisons are not directly equivalent to the four-tier intensity scoring (negative, weak, moderate, strong) applied here. Liu et al. examined 1153 primary prostate cancers from the Canary Prostate Cancer TMA cohort by IHC and reported associations between high TROP2 expression, higher Gleason grade, and shorter overall survival in univariate analysis, although TROP2 did not reach independent significance in multivariate models [20]. Trerotola et al. described increased TROP2 expression in tumors with extracapsular extension on a TMA of 148 cancers [18]. Hsu et al. found high TROP2 expression to represent an independent predictor of biochemical recurrence on a TMA of 234 primary prostate cancers [19].

Because of the large number of cases analyzed, maximal standardization, and the extensive previous validation of our IHC assay, we are confident that our observation of reduced but not elevated TROP2 expression going along with tumor progression reflects the reality. This notion is additionally supported by the significant further decrease in TROP2 expression in the cohort of 250 recurrent prostate cancers which may represent the most relevant finding of this study. That markedly lower TROP2 levels in recurrent tumors were not only seen in the overall cohort, but also in a comparison with high-grade primary cancers demonstrates that lower TROP2 in recurrent cancers is not caused by a different grade composition in recurrent cancers but likely reflects further tumor dedifferentiation which regularly accompanies tumor progression. Obvious “molecular progression” also highlights a significant limitation of the Gleason grade which has been designed for assessing primary prostate cancers and does not include parameters for the quantification of further progression occurring in prostate cancer under therapy. Molecular progression in high-grade prostate cancer is characterized by a reciprocal pattern of protein expression changes. Markers physiologically expressed in prostate epithelium, including androgen receptor (AR) [26], prostate-specific antigen (PSA) [29], prostate-specific membrane antigen (PSMA) [30] and, as shown here, TROP2, are progressively lost. Proteins absent in normal prostate epithelium may be aberrantly gained, including neuroendocrine markers or SPINK1, a marker of a distinct ERG-fusion-negative molecular subtype, alongside increased proliferative activity.

The availability of data from numerous earlier studies is a strength of our prostate cancer TMA. In this study we compared our findings with data on ERG fusion because this represents one of the most common genomic alterations in prostate cancer [28]. About 50% of prostate cancers, particularly those of young patients, contain fusions connecting the androgen-regulated *TMPRSS2* gene to the transcription factor ERG [23,31]. These rearrangements result in an androgen-dependent overexpression of ERG [32] which subsequently alters the expression of more than 1600 genes in prostate epithelial cells [33]. The finding that reduced TROP2 expression was not tightly linked to ERG expression is further evidence for the concept that TROP2 downregulation may represent a random feature of dedifferentiated cells rather than the deregulation of a specific pathway.

While our data demonstrate that measuring TROP2 expression cannot support the assessment of the aggressive potential of primary prostate cancers, they are in line with a role of TROP2 as a suitable therapeutic target in this tumor entity. This is further supported by objective responses or disease control in some heavily pretreated mCRPC patients undergoing SG therapy in individual case reports [16] and early-phase [15] and basket-trial [8] cohorts. Notably, pilot data from the phase II trial combining SG with an androgen receptor signaling inhibitor suggest that this combination may help restore sensitivity to AR-directed therapy [15], and further evaluation of SG-based combination strategies in prostate cancer appears warranted. Based on the substantial reduction in TROP2 expression in many recurrent prostate cancers, it appears possible that the quantification of TROP2 immunostaining can be of use for potentially identifying patients most likely to benefit from these treatments, although this remains to be demonstrated in prospective, treatment-annotated cohorts.

Importantly, reduced TROP2 in recurrent disease does not preclude a therapeutic role: the majority of recurrent cancers (approximately 89%) retained moderate or strong TROP2 expression and would thus be expected to remain accessible to TROP2-directed therapy, whereas the minority with weak or absent staining (10.8%) represent those in which TROP2 measurement could help to avoid treatment that is unlikely to be effective. The clinical value of TROP2 immunohistochemistry in recurrent disease therefore lies in patient selection rather than in the reduction in expression per se.

Our study has several limitations. First, it is descriptive and does not define the mechanism by which TROP2 expression is reduced in recurrent disease, although the parallel loss of other prostate-lineage proteins is consistent with progressive tumor dedifferentiation. Second, while the primary cohort is large, the recurrent cohort (258 samples, 250 evaluable) is comparatively small, was derived from archived diagnostic material, and lacked consistent annotation of prior systemic and androgen-deprivation therapy; these factors may influence TROP2 expression—for example, androgen-deprivation therapy and androgen-receptor pathway inhibition may promote lineage dedifferentiation and thereby reduce the expression of prostate-lineage proteins, including TROP2—and may render the recurrent cohort a biologically selected population. More broadly, detailed characterization of the recurrent tumors—including castration-sensitive versus castration-resistant status, prior chemotherapy, local versus metastatic recurrence, and neuroendocrine differentiation—was not available, and because these features critically shape tumor biology, the mechanisms underlying the reduction in TROP2 in recurrent disease cannot be determined from the present data. Third, TROP2 was evaluated on single 0.6 mm tissue cores, which may incompletely capture intratumoral heterogeneity, particularly in advanced and recurrent tumors. Fourth, immunohistochemical scoring was semi-quantitative; although staining was performed in a single standardized batch and scored by experienced pathologists, formal interobserver agreement was not determined and automated image-based quantification was not applied. In addition, the four-tier categorical scoring provides only limited resolution; for antibody–drug conjugate therapies, differences in target density within the ‘strong’ category may be clinically relevant, and a continuous score such as an H-score, or automated digital image analysis, could provide finer biological resolution. Fifth, the available clinical endpoint was biochemical (PSA) recurrence rather than metastasis-free, cancer-specific, or overall survival. Moreover, we note that the association between reduced TROP2 expression and early PSA recurrence was limited to ERG-positive cancers and that it was not adjusted for multiple comparisons; these subgroup analyses should therefore be regarded as hypothesis-generating and require independent validation.

Sixth, TROP2 protein levels were not correlated with TACSTD2 mRNA expression, although the association with ERG status was assessed. Finally, the cross-sectional design precludes evaluation of intra-patient TROP2 dynamics from primary to recurrent disease, and the findings were not confirmed in an independent validation cohort. Together with the absence of patients treated with TROP2-directed agents in this series, these limitations mean that as no treatment-response data are presented in this study, the predictive value of TROP2 measurement for response to TROP2-targeted therapy remains to be established in prospective studies.

## 5. Conclusions

In summary, TROP2 is uniformly and strongly expressed in primary prostate cancer but is frequently reduced in recurrent disease. While TROP2 expression is unsuitable as a prognostic marker in primary tumors, its frequent retention—even at high levels—in recurrent cancers under therapy suggests a possible role as a suitable therapeutic target in progressive disease. Given the high variability of TROP2 expression levels in recurrent prostate cancers including 10.8% with low or absent staining, TROP2 IHC may help to identify the most suitable candidates for SG therapy in this setting. This hypothesis warrants prospective evaluation.

## Figures and Tables

**Table 1:** Pathological and clinical data of the arrayed prostate cancers. Percent in the column “Biochemical relapse among categories” refers to the fraction of samples with biochemical relapse within each category.

Variable	Category	No. of Patients (%)	
		Study Cohort on TMA (*n* = 17,747)	Biochemical Relapse Among Categories
Follow-up (mo)	n	14,464 (81.5%)	3612 (25.0%)
	Mean	56.3	–
	Median	48	–
Age (y)	≤50	433 (2.4%)	66 (15.2%)
	51–59	4341 (24.5%)	839 (19.3%)
	60–69	9977 (56.4%)	2073 (20.8%)
	≥70	2936 (16.6%)	634 (21.6%)
Pretreatment PSA (ng/mL)	<4	2225 (12.6%)	313 (14.1%)
	4–10	10,520 (59.6%)	1696 (16.1%)
	10–20	3662 (20.8%)	1043 (28.5%)
	>20	1231 (7.0%)	545 (44.3%)
pT stage (AJCC 2002)	pT2	11,518 (65.2%)	1212 (10.5%)
	pT3a	3842 (21.7%)	1121 (29.2%)
	pT3b	2233 (12.6%)	1213 (54.3%)
	pT4	85 (0.5%)	63 (74.1%)
Gleason grade	≤3+3	3570 (20.3%)	264 (7.4%)
	3+4	9336 (53.0%)	1436 (15.4%)
	3+4 Tert.5	798 (4.5%)	165 (20.7%)
	4+3	1733 (9.8%)	683 (39.4%)
	4+3 Tert.5	1187 (6.7%)	487 (41.0%)
	≥4+4	999 (5.7%)	531 (53.2%)
pN stage	pN0	10,636 (89.4%)	2243 (21.1%)
	pN+	1255 (10.6%)	700 (55.8%)
Surgical margin	Negative	14,297 (80.8%)	2307 (16.1%)
	Positive	3388 (19.2%)	1304 (38.5%)

**Table 2:** Association between TROP2 immunostaining and clinicopathological parameters in 12,807 primary prostate cancers.

Variable	Category	n Evaluable	TROP2 MSVA-733R IHC Result			*p* Value
			Weak (%)	Moderate (%)	Strong (%)	
All cancers		12,807	2	3.5	94.5	
Tumor stage	pT2	7744	1.6	3	95.3	0.0011
	pT3a	2975	2.2	3.8	94	
	pT3b-4	1896	2.7	4.1	93.2	
Gleason grade	≤3+3	2120	1.4	3.4	95.2	<0.0001
	3+4	6769	1.7	2.8	95.8	
	3+4 Tert.5	631	2.1	2.7	95.2	
	4+3	1279	2.7	4.6	92.7	
	4+3 Tert.5	972	2.6	5.2	92.3	
	≥4+4	793	3.8	6.1	90.2	
Quantitative Gleason	3+4 ≤5%	1662	1.7	2.5	95.8	<0.0001
	3+4 6–10%	1601	1.5	2.2	96.2	
	3+4 11–20%	1489	1.4	2.2	96.4	
	3+4 21–30%	761	1.4	2.4	96.2	
	3+4 31–49%	849	2	5.1	92.9	
	4+3 50–60%	601	2.1	2.7	95.2	
	4+3 61–80%	460	2.2	3	94.8	
	4+3 >80%	117	3.6	6.1	90.4	
Lymph node metastasis	N0	7848	2	3.4	94.6	0.2423
	N+	1034	2.8	3.4	93.8	
Preop. PSA level (ng/mL)	<4	1466	1.6	3.3	95.1	0.1038
	4–10	7352	1.7	3.2	95.1	
	11–20	2746	2.3	3.9	93.7	
	>20	1026	2.6	3.4	94	
Surgical margin	negative	9899	1.8	3.3	94.9	0.1623
	positive	2720	2.4	3.6	94.1	

**Table 3:** Multivariate Cox regression analysis of TROP2 expression and biochemical (PSA) recurrence in prostate cancer. Four scenarios were calculated for the overall cohort and for ERG-negative and ERG-positive subgroups. Scenario 1: all postoperative parameters; Scenario 2: excluding pN status; Scenario 3: preoperative setting with prostatectomy Gleason grade; Scenario 4: preoperative setting with initial biopsy Gleason grade derived from a multitude of institutions.

Tumor Subset	Scenario	n Analyzable	*p*-Value							
			Preoperative PSA-Level	pT Stage	cT Stage	Gleason Grade Prostatectomy	Gleason Grade Biopsy	pN Stage	R Status	TROP2- Expression
All cancers	1	6879	<0.0001	<0.0001	-	<0.0001	-	<0.0001	<0.0001	0.0713
	2	10,135	<0.0001	<0.0001	-	<0.0001	-	-	<0.0001	0.0417
	3	9968	<0.0001	-	<0.0001	<0.0001	-	-	-	0.0331
	4	8390	<0.0001	-	<0.0001	-	<0.0001	-	-	0.3554
ERG-negative cancers	1	2951	<0.0001	<0.0001	-	<0.0001	-	<0.0001	0.1542	0.0881
	2	4335	<0.0001	<0.0001	-	<0.0001	-	-	0.0029	0.2667
	3	4275	<0.0001	-	<0.0001	<0.0001	-	-	-	0.0843
	4	3558	<0.0001	-	<0.0001	-	<0.0001	-	-	0.2131
ERG-positive cancers	1	2431	0.0007	<0.0001	-	<0.0001	-	0.0084	0.0002	0.4052
	2	3631	<0.0001	<0.0001	-	<0.0001	-	-	<0.0001	0.0220
	3	3554	<0.0001	-	<0.0001	<0.0001	-	-	-	0.3974
	4	3013	<0.0001	-	<0.0001	-	<0.0001	-	-	0.2138

## Data Availability

All data supporting the findings of this study are available within the article; additional data are available from the corresponding author upon reasonable request.

## Supplementary Materials

The following supporting information can be downloaded at https://www.mdpi.com/article/10.3390/cancers18142359/s1. Figure S1: representative examples of weak-positive TROP2 immunostaining in prostate cancer tissue microarray spots (A–O); Figure S2: representative examples of moderate-positive TROP2 immunostaining in prostate cancer tissue microarray spots (A–O); Figure S3: representative examples of strong-positive TROP2 immunostaining in prostate cancer tissue microarray spots (A–O).

## Abbreviations

The following abbreviations are used in this manuscript:

ADC	Antibody–drug conjugate
AR	Androgen receptor
CRPC	Castration-resistant prostate cancer
ERG	ETS-related gene
FISH	Fluorescence in situ hybridization
IHC	Immunohistochemistry
IWGAV	International working group for antibody validation
mCRPC	Metastatic castration-resistant prostate cancer
PSA	Prostate-specific antigen
PSMA	Prostate-specific membrane antigen
PTEN	Phosphatase and tensin homolog
RP	Radical prostatectomy
SG	Sacituzumab govitecan
TACSTD2	Tumor-associated calcium signal transducer 2
TMA	Tissue microarray
TMPRSS2	Transmembrane serine protease 2
TROP2	Trophoblast cell surface antigen 2
