# Supplementary material for: TROP2 Is Uniformly Expressed in Primary Prostate Cancer but Frequently Reduced in Recurrent Disease
Source: Cancers (Basel). 2026 Jul 22;18(14):2359. doi: 10.3390/cancers18142359 (PMC13406205; doi:10.3390/cancers18142359)

Supplementary Figure S2. TROP2 immunostaining in prostate cancer. The panels show representative examples of moderate-positive TROP2 immunostaining in prostate cancer tissue microarray spots (A-O).

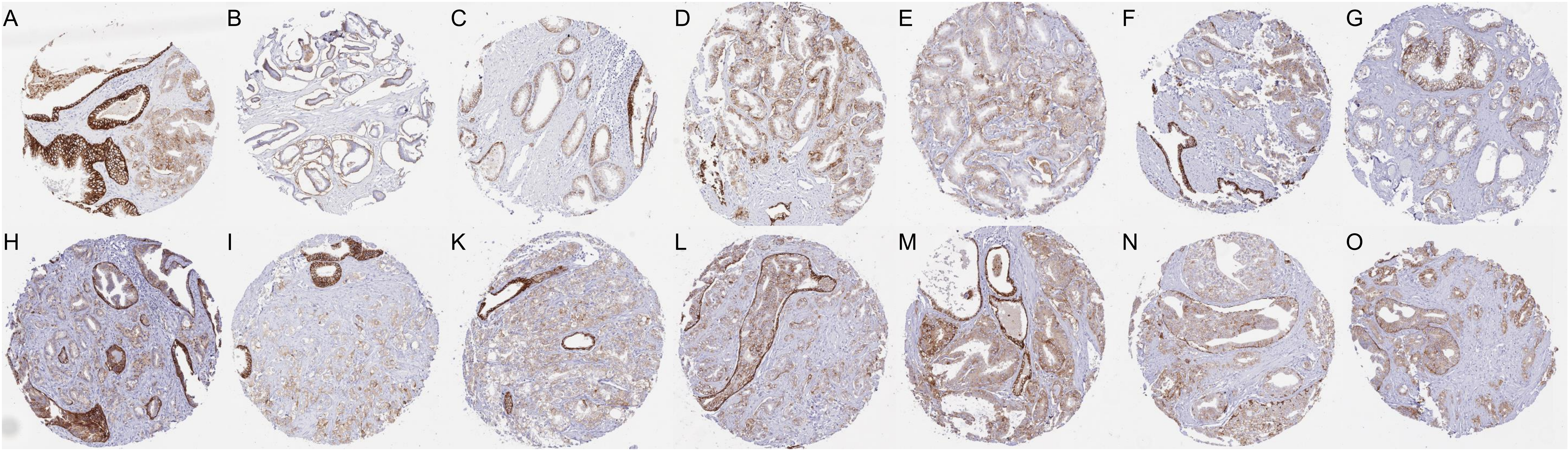

Supplement: Supplementary file 1 [file cancers-18-02359-s001.zip › Supplementary Figure S2 TROP2 moderate-positive.pdf]
